# Personalizing guidelines for diabetes management: twilight or dawn of the expert?

**DOI:** 10.1186/1741-7015-11-161

**Published:** 2013-07-10

**Authors:** Stavroula A Paschou, Richard David Leslie

**Affiliations:** 1 Department of Diabetes & Endocrinology, Hellenic Red Cross Hospital, Athens, 11526, Greece; 2 Department of Diabetes, St Bartholomew’s Hospital, London, EC1A 7BE, UK

**Keywords:** Type 2 diabetes, Guidelines, Personalised treatment, Diabetes management, Patient-centered therapy

## Abstract

**Background:**

This opinion article on the management of type 2 diabetes considers the old and new format of guidelines and critical changes in the character of such guidelines. We highlight limitations of the guidelines and make recommendations for how treatment can be more personalised.

**Discussion:**

Published guidelines for the management of adult-onset non-insulin requiring diabetes have adopted a formulaic approach to patient management that can be overseen centrally and delivered by personnel with limited training. Recently, guidelines have taken a patient-centered, multiple risk-factor approach. Importantly, local funding issues are considered, but drive the final action and not the decision-making process. The nature of the disease can be determined by laboratory tests, including screening for diabetes-associated autoantibodies. The strategy remains step-up, with intensification of drug or insulin dose. As with past guidelines, there is an assumption that in each patient with type 2 diabetes, metformin is used initially, but targets and therapies then veer in different directions to create a matrix of options based on the features and responses of each individual. Factors to consider include: (A)ge, (B)ody weight, (C)omplications and co-morbidities, Diabetes (D)uration and (E)xpense, but also patient preference and patient response.

**Summary:**

Guidelines for the management of type 2 diabetes have important limitations and a patient-centered, multiple target, multiple therapy approach is proposed.

## Background

Ancient Greek Hippocrates (c. 460 BC to c. 370 BC) advised physicians for patients: *‘ωϕελέειν ή μη βλάπτειν’, ‘to do good or to do no harm'.* Modern physicians take the Hippocratic Oath in its various forms, but remain committed to the risk-benefit ratio implicit in this statement, to the overall benefit of their patients.

Diabetes is a complex disease characterized by deterioration of glycaemia and co-morbidity. In adult patients, the comorbidity is often the result of a risk factor spectrum associated with a high proportion of cases and broadly identified as the Metabolic Syndrome, with the clustering of obesity, hypertension and dyslipidemia, as well as hyperglycemia [1-5]. In contrast to disease nosology, therapies have no identity, only a mode of action, a list of side-effects and a financial cost. Today we have many drugs in our armamentarium to treat type 2 diabetes, but many of these drugs are relatively new. So, deciding which direction to take for any given patient, even whether to use drugs at all, is akin to standing on shifting sands. That decision is further complicated by the delicate risk-benefit balance, highlighted by recent intervention trials (ACCORD, ADVANCE, VADT) [6-8].

In this opinion article we will tackle these issues as handled by the rash of recent guidelines. In one sense this dawn of the guidelines could spell the twilight of the professional, for guidelines are presented as simple guides to patient management to be applied by health care providers who are not experts in diabetes management. Such guidelines raise the important question as to whether diabetes management can be formulaic or whether it must be professional and patient-centered. Importantly, this review is not a guideline. Indeed, our aim is to highlight the limitations of guidelines. In short, it is to herald a return to the personal professional care provided by an expert, without recourse to centralized broad strategies.

## Discussion

### Overarching strategy

Patients with diabetes are at risk of macrovascular and microvascular disease [7,8]. Strikingly, the risk factors for these complications are similar. However, factors associated with macrovascular disease tend to be most strongly associated with insulin resistance and the metabolic syndrome, that is, obesity, dyslipidemia and hypertension while hyperglycemia is the dominant factor associated with microvascular disease. It follows that the management of diabetes resolves around the management of multiple risk factors, notably when treating patients with type 2 diabetes [1-5]. Therapeutic plans for managing non-insulin requiring diabetes are: anecdotal and historical (often), evidence-based (infrequently) or outcome based (rarely). Such plans usually offer a broad guide for the generality of patients, but recently encompass a more ‘personalised’ approach which is targeted to specific qualities of each individual [9,10]. Since there are multiple risk factors to consider for each individual and we are focusing here on hyperglycemia alone, it follows that the strategy to be employed is complex. Two decisions must be made initially: the appropriate target of therapy and the appropriate therapeutic strategy.

### Appropriate therapeutic targets

Hyperglycemia is usually determined using glycated haemoglobin (HbA1c), an index of average blood glucose levels over two to three months, determined by the percentage of haemoglobin with an adduct of glucose. Therapeutic targets should vary from patient to patient. For optimum diabetes control an HbA1c value of less than 6.5% (48 mmol/mol) is widely proposed [1-5]. However, for patients who are older, of longer disease duration or with complications, such strict targets are not validated and there is a move towards a more flexible approach for a given individual – so-called personalized medicine [9,10]. The target HbA1c will then determine the nature of the therapy and that target can be modified according to both diabetes duration and co-morbidities.

#### Targets can determine therapy

The glucose-lowering effectiveness of agents varies, so the degree of HbA1c reduction sought will impact the decision as to which agent to use. Insulin aside, the efficacy is high for metformin, sulfonylureas (SUs), thiazolidinediones (glitazones) and glucagon-like peptide-1 (GLP-1) agonists (expected HbA1c reduction approximately 1.0 to 1.5%) and, generally, lower for meglitinides, dipeptidyl peptidase-4 (DPP-4) inhibitors, acarbose, colesevelam and bromocriptine (approximately 0.5 to 1.0%) (Table 1). Historically, older drugs have typically been tested in clinical trial participants with higher baseline HbA1c, so, without direct comparisons, it is difficult to be certain that SUs, for example, really are more or less effective that DPP-4 inhibitors or GLP-1 agonists; but these latter are consistently found not to be inferior to SUs, with the added benefit of no weight gain, even weight loss, without hypoglycemia [1,11-18]. Agents with more modest effects, but little or no side-effects, could be considered when treating individuals with impaired glucose tolerance, to limit progression to frank diabetes, for example, acarbose, though it is prone to cause flatulence [9].

**Table 1:** **Important characteristics of main glucose**-**lowering agents**

	**HbA1C reduction (%)**	**Hypoglycemia**	**Weight**	**Use in heart failure**	**Use in renal failure**	**Stimulation of insulin secretion**	**Increase of insulin sensitivity**	**Durability of action**	**Main side effects**	**Outcome benefit**	**Relevant references**
Metformin	1.0 to 1.5	-	-	+/-	-	-	+	~↑	Gastrointestinal	+	[1,2,10,12,32,37,54,55],[60,65]
									Lactic acidosis		
Sulfonylureas	1.0 to 1.5	+	↑	-	+/-	+	-	↓	Hypoglycemia	+/-	[1,2,10,12,32,37,47,76]
									Weight gain		
Meglitinides	0.5 to 1.0	+	↑	-	+	+	-	↓	Hypoglycemia		[1,2,10,12,46,76]
									Weight gain		
Thiazolidinediones	1.0 to 1.5	-	↑	-	+	-	+	~↑	Weight gain		[1,2,10,12,37,58-60,63,64]
									Oedema/Heart failure		
GLP-1 receptor agonists	1.0 to 1.5	-	↓	+	+/-	+	-	?	Gastrointestinal	?	[10-18,72,73,75,76]
									? Pancreatitis		
DPP-4 inhibitors	0.5 to 1.0	-	-	+	+/-	+	-	?	? Pancreatitis	?	[10-12,61,64,73,75,76]
Insulin therapy	>1.0	+	↑	+/-	+			↑	Hypoglycemia	+	[6,7,10,20-22,32,50-52]
									Weight gain		
									?Mitogenic effects		

*DDP-4* dipeptidyl peptidase-4, *GLP-1* glucagon-like peptide-1, *HbA1C* glycated haemoglobin, *~* approximately.

Insulin is widely used to treat type 2 diabetes because numerous studies indicate that it works. However, it carries a risk of both hypoglycemia and weight gain, two critical issues in assessing the value of any given therapy. The fall in HbA1c comes at a cost of rising hypoglycemia-risk, and analogues of long-acting and short-acting insulin reduce nocturnal hypoglycemia and post-prandial glucose, respectively, without benefiting HbA1c levels. GLP-1 agonists are as effective as insulin at reducing HbA1c, with the additional benefit of weight loss without hypoglycemia, but then a proportion of cases develop nausea and acute pancreatitis is, possibly, an additional, albeit very small, risk [17]. Initial insulin therapy for type 2 diabetes patients is unusual unless the patient is markedly hyperglycemic and/or symptomatic [3,9,10,12]. Patients with ketosis prone diabetes (KPD) are in the category of those who present with ketocidosis but after initial insulin therapy can stop insulin and revert to tablet treatment [19]. The ORIGIN trial failed to find a role for low-dose insulin therapy early in the course of type 2 diabetes, in that insulin treatment did not reduce cardiovascular risk [20].

Approaches to insulin therapy include three regimes: a single injection of basal insulin, multiple prandial insulin injections and two injections of biphasic insulin. The only trial comparing these regimens concluded that basal and prandial insulin each provide better glucose control than the biphasic insulin regimen, while basal insulin benefits from fewer hypoglycemic episodes and less weight gain [21]; however, many patients taking basal insulin were also taking prandial insulin in this three-year comparison study. Comparison at one year, when patients were on their assigned insulin only, indicated that basal insulin was inferior in lowering HbA1c to prandial insulin or mixed insulins given twice daily [21]. Basal insulin alone tends to be used initially, but twice daily injections using either pre-mixed insulin or a long-acting insulin with a single prandial injection are a reasonable second-line of therapy, short of progressing to four daily injections [21].

##### Targets modified by co-morbidities

While target HbA1c levels can be specified in guidelines, these levels are modified by comorbidities, so it is important to take other diseases into account. In patients without established complications and with short disease duration, both the risk of microvascular and macrovascular disease is reduced by lowering HbA1c (every approximately 1% HbA1c reduction is associated with a 25% relative risk reduction for microvascular disease progression) [22-30]. Targets are also affected by diabetic retinopathy, which is a serious complication of diabetes. Recommendations tend to suggest obtaining better glucose control in the presence of retinopathy than in those without retinopathy [25,26].

Cardiovascular complications are important considerations when patients are given HbA1c-lowering therapy. A meta-analysis of cardiovascular outcomes in major trials suggested that every approximately 1% HbA1c reduction is associated with a 15% relative risk reduction in non-fatal myocardial infarction, but without benefit on stroke or all-cause mortality [28] while the same HbA1c reduction is associated with a 37% risk reduction in microvascular complications [26].

##### Targets and duration of disease

Disease duration should be considered when defining HbA1c targets. Early strict glycaemic control prevents micro- and macrovascular complications [22]. Analyses of recent intervention trials in type 2 diabetes indicate that the shorter the disease duration the greater the cardiovascular protection offered by strict glycemic control. Once disease duration is more than 10 to 12 years, that beneficial effect may be lost, may even become detrimental [27-30]. On the other hand, *post hoc* analysis of some data suggests that benefits of glycaemic control are likely to occur in patients before the development of clinical cardiovascular disease regardless of the duration of their disease [30]. In young patients, reducing the long-term risk of complications, while ensuring optimal energy metabolism, demands more strict HbA1c targets. Therefore, we should achieve lower targets and get there faster in younger patients [9] while we should aim for safer targets and achieve them more slowly in older patients [9].

Advanced microvascular complications are difficult to reverse and, by implication, the same is true of established macrovascular disease. Therefore, patients with major micro- and macrovascular complications should not be given strict HbA1c targets. However, in patients with background retinopathy, irrespective of other vascular disease, progression of retinopathy can be limited by controlling HbA1c so, in these patients, a strict HbA1c target could be beneficial [25,26]. Less strict HbA1c goals can be considered for patients with a history of severe hypoglycemia, advanced complications, co-morbid diseases and when life expectancy is limited. When it is difficult to achieve targets, despite intensive efforts, it is important to modify the treatment goals, remembering that the risk of complications with increasing HbA1c is exponential, so that a 1% reduction of HbA1c from 10.0 to 9.0% (84 to 75 mmol/mol) should be more effective than an HbA1c reduction from 8.0 to 7.0% (64 to 53 mmol/mol) [26]. Taking all these factors into account, it is important to analyze comorbidities in individual patients in order to determine the optimum HbA1c target levels.

### Appropriate therapy

As is the case for targets of therapy, so the best drug treatment will vary between patients.

#### Step-up or step-down

Guidelines tend to be based on: step-up therapy by drug numbers; step-up therapy by intensification of drug action; single risk factor (for example, hyperglycemia or hypertension, but not both); specific and universal targets (for example, HbA1c 7.0%); and the consideration of type 2 diabetes as a single disease [9,31-33]. However, the stepwise addition of oral agents and/or insulin contrasts with other diseases, such as rheumatoid arthritis or gout, in which initial management is intense and, subsequently, more modest – a step-down approach [34,35]. We adopt a step-down approach when a patient presents with marked hyperglycemia (for example, HbA1c ≥9.0%) in which case insulin, unless poorly tolerated or contraindicated, is combined with or without metformin. However, some therapies, other than insulin, can be effective even when HbA1c is high, for example, GLP-1 agonists (for options see Table 1).

At least one recent study has taken a step-down approach to initial intensive therapy, using a combination of drugs at low dose, but final results of that study are awaited [31]. Some 30 years ago in the USA only insulin was available for treatment of diabetes for those unresponsive to diet and exercise alone. Metformin was tarred by association with phenformin, and SUs had been vilified in the, now broadly discredited, University Diabetes Group Program [36]. The strategic problems we now face have come about through the success of the pharmaceutical industry. The medical profession must resolve how best to utilize this array of drugs and injectables.

#### Step-up hierarchy

Management of type 2 diabetes, once life-style intervention has failed, usually involves the introduction of metformin [9,10]. Some people still prefer insulin, even at this stage. As drugs fail, other agents are introduced, historically SUs and then insulin but the broad range of alternative therapies has led to a more flexible approach towards second and third line therapy.

Durability of drug action is an important consideration in the management of type 2 diabetes, but is not broadly considered in guidelines owing to the lack of alternatives [37]. The reduction in beta-cell function, both before and after diagnosis of type 2 diabetes, is the likely cause of the progressive rise in HbA1c following diagnosis [37]. In the UK Prospective Diabetes Study (UKPDS), all therapies (insulin, metformin, SUs, lifestyle) were associated with an increasing HbA1c. In other words, all drugs failed in time, of which SUs, initially the most effective, were the worst [26]. It remains possible that new drugs, such as glitazones, GLP-1 analogues and DPP-4 inhibitors may preserve beta cell function, although clear proof of this is awaited [9,37].

#### Multiple targets and outcomes

Since type 2 diabetes involves multiple risk factors, it follows that therapy also involves multiple targets, multiple potential outcomes and multiple drugs to achieve those targets for each individual. Guidelines rarely consider the interrelationship between multiple therapeutic options, but do consider the list of modifiable risk factors including: blood pressure and lipid therapy, anti-platelet treatment and smoking cessation. For example, fenofibrate, a lipid-lowering agent, may also be beneficial in limiting the progression of retinopathy [38].

Most drug trials focus on lowering of blood glucose, weight loss and hypoglycemia, whilst omitting outcome in terms of macrovascular and microvascular disease [13-18]. Therefore, where these results are available from major outcome studies (for example, UKPDS and Diabetes Control and Complications Trial (DCCT)) they have dominated current management [39-42]. Central to the successful implementation of each treatment has been the reduction of HbA1c, although both hypoglycemia risk and patient preference are often ignored beyond severe hypoglycemia. For example, the most successful therapy to reduce blood glucose is insulin. Yet insulin is not recommended as the initial treatment, unless hyperglycemia is severe, by implication because of the side-effects, management resources and patient preference [9,10]. Similarly, SUs are initially more effective than metformin, but the latter is more widely used at diagnosis, probably because of the side-effects of SUs (although the frequency of side-effects is higher with metformin) and the suggestion that metformin has a beneficial effect on all-cause mortality – at least, in the absence of SUs [22,26].

#### Non-insulin requiring diabetes: multiple diseases?

The heterogeneity of diabetes is such that it is difficult to be certain of the cause of the disease in any given patient without recourse to laboratory tests. Non-insulin dependent diabetes is a diagnosis by exclusion, that is by exclusion of insulin-dependent diabetes. There is substantial evidence, however, that about 10% of patients, both European and Chinese, presenting in adulthood with diabetes that does not, at least initially, require insulin treatment, having diabetes-associated autoantibodies and the HLA genetic susceptibility and protection found in type 1 diabetes [43,44]. Moreover, other forms of diabetes, while rare, exist and can present in adult life including: ketosis prone diabetes (KPD), maturity onset diabetes of young onset (MODY) and haemochromatosis, as well as other forms of secondary diabetes, for example, following steroid treatment [9,19,42]. The heterogeneity of diabetes should be considered when selecting the most appropriate therapy for individual patients.

SUs are probably contraindicated in patients with adult-onset autoimmune diabetes, including latent autoimmune diabetes of adults (LADA), due to the marked lack of durability of their action by comparison with insulin [45]. Anecdotal evidence supports the use of DPP-4 inhibitors and GLP-1 agonists in non-insulin requiring adult-onset autoimmune diabetes, but formal comparisons are awaited. Meglitinides (or glinides) are short-acting secretagogues that stimulate insulin release through similar mechanisms to SUs, but may be associated with less hypoglycemia [46] (Table 1). However, they require more frequent dosing. A particular indication, due to their modest and short action, is for patients with type 3 MODY in whom the risk of hypoglycemia is reduced [46]. Patients with steroid-induced diabetes tend to have post-prandial hyperglycemia, but not fasting hyperglycemia; they may benefit from short-acting insulin analogues or SUs and if steroid therapy is transient as with asthma treatment then DDP-4 inhibitors could be preferable, as the risk of hypoglycemia, as the glucose falls and treatment is withdrawn, is less than for insulin or SUs.

#### Therapy and co-morbidities

The universal benefit of reducing blood glucose, irrespective of the agent, indicates a generic effect, likely true for all glucose-lowering treatments. So, as outcome studies become available, we will have to modify whatever position we currently take on the newer therapies. Co-existent diseases which impact therapeutic decisions include renal disease and macrovascular disease.

##### Macrovascular disease

Macrovascular disease can impact the use of certain drugs. Recommendations for those with cerebrovascular disease in general match those with cardiovascular disease; while SUs and glinides should be avoided and glitazones have a complex role, all other treatment options can be used to control blood glucose as appropriate. SUs (and glinides) have been reported to reduce myocardial blood flow and increase early mortality after acute ischaemia [46]. In UKPDS, SUs confounded the mortality benefit of metformin [21]. Metformin is contraindicated in patients within two weeks after stroke (Glycaemia in Acute Stroke (GLIAS), UK Glucose Insulin in Stroke Trial (GIST-UK), Diabetes Mellitus Insulin Glucose Infusion in Acute Myocardial Infarction (DIGAMI))[47-52]. Metformin bears the additional risk of lactic acidosis, especially in patients with recent myocardial infarction but it is not contraindicated in patients with cardiovascular disease; indeed, recent studies have suggested that it may be beneficial [53-55]. Glitazones should not be used in Stage III-IV heart failure as they can exacerbate the condition. Preferred drugs in heart failure include metformin (NYHA Stage I-II), acarbose and GLP-1 analogs/DPP-4 inhibitors, although the evidence base to recommend the latter is weak [56-61]. Of the glitazones, pioglitazone remains an agent whose precise role is to be determined [61,62] but which appeared to have a modest benefit on cardiovascular events as a secondary outcome in one large trial (ProActive) [62], although with weight gain as a substantial side-effect and bladder cancer as a potential problem [63] (Table 1). Rosiglitazone is no longer available due to a postulated, and disputed, increased myocardial infarction risk [64].

##### Impaired renal function

When renal function is impaired, drugs cleared by the kidney may not be cleared appropriately, so that blood levels may increase. Metformin and liraglutide are contraindicated when the glomerular filtration rate (GFR) <60 ml/min. DPP-4 inhibitors when GFR <50 ml/min are broadly contraindicated (or the dose should be adjusted) but linagliptin can be used at all stages of renal dysfunction [65,66]. Exenatide and SUs are contraindicated when GFR <30 ml/min. Acarbose is contraindicated when GFR <25 ml/min. Insulin is generally recommended when renal failure is advanced, linagliptin being a potential alternative [9,10,66].

In summary, co-morbidities can direct drug use and drug action as well as the risk of drug side-effects. These potential effects will vary from one drug to another and between patients to influence therapeutic decisions.

### Individualised targets and therapies

We have already discussed the need to individualize therapeutic targets based on age, complications and co-morbidities and disease duration; the same applies to therapies. For all medications, consideration should also be given to overall tolerability. Even occasional hypoglycemia may be devastating, if severe, or merely irritating, if mild [67].

Gastrointestinal side effects may be tolerated by some, but not others. Fluid retention may pose a clinical but also, an aesthetic, problem. Substantial individual variation in side-effects, as well as beneficial effects of all agents, influences our approach to the individual case depending on the patient’s response. At least one expert has proposed drug-ranging trials to determine the best positive/negative ratio for each agent in any given patient [68]. Adult patients who live alone or have occupations which preclude a degree of risk of hypoglycemia, for example, the elderly, taxi drivers and sportsmen, should avoid insulin, SUs and glinides and favor metformin and DPP-4 inhibitors [69,70]. Pregnancy or the wish to get pregnant will also preclude the newer drugs, as well as statins, in that the risk associated with them to the fetus is unknown [9,10,71].

Finally, those patients who are obese will favor agents that are weight neutral or reduce weight, such as metformin and GLP-1 agonists (Table 1). Because weight gain can be associated with increasing risk of cardiovascular disease, it is a side-effect physicians also wish to avoid. A recent study showed little difference in a once daily versus once weekly preparation of GLP-1 agonist in terms of improved HbA1c and tolerability but raised interesting questions as to the perceived optimum therapy since the once weekly therapy did not achieve as low a HbA1c [72]. Indeed, the need for individualized therapy is highlighted by these GLP-1 agonists which are associated in some patients with initial gastrointestinal side effects (nausea and vomiting), so that a proportion of patients cannot tolerate them, nor do they work in at least 25% of cases [73]. Some drug classes have greater efficacy in obese than non-obese individuals, for example, glitazones [73,74] while others are preferable in marked obesity, for example, GLP-1 agonists [75]. If body weight is successfully reduced, anti-diabetic medication often has to be adjusted [76]. Bariatric surgery, not discussed here, remains an important option when all else fails [77,78]. In summary, the need to individualize therapy based on age, complications, co-morbidities and disease duration, extends to the individual’s response to each therapy and their perception of each therapy, for example, the fear of injections as compared with the fear of insulin injections.

#### Multifaceted interaction

Whilst, at first sight, the doctor might make a decision based on the details about the patient, in reality two other factors are involved in that decision. First, the patients themselves bring with them a raft of prejudices and concerns, fears and constraints, which may have to be carefully elicited. Notable among these fears are the fear of insulin. That fear stems from the fear of hypoglycemia and of injections, but also from concerns about weight gain or from historical issues, such as relatives who started insulin treatment and fared badly. Second, the funding agencies often seek to determine, by constraint or coercion, which therapies are available or can be used. Given the substantial variation in resources around the world, both in terms of access to drugs as well as the cost of drugs, it follows that discussion of costs could only be parochial and will be broadly avoided here. But the cheaper drugs are drugs with a long-history (metformin, SUs and human insulin), while more expensive drugs are those only recently available (the rest). It follows that outcome studies, which are available for ‘historical’ forms of treatment, provide a justification for their use.

## Summary

The definition both of the target of HbA1c as well as therapeutic options and individual responses to therapy can guide clinicians towards more effective, efficient and safe treatment of type 2 diabetes. In this article we outlined the matrix of options available to the physician and highlighted factors critical to decision-making, without setting out a precise plan, in the spirit of ‘personalized’ therapy. It is our contention that the complexity of the matrix of options precludes rigid guidelines. We appreciate that this approach is less cost-effective when compared with guidelines, which can be followed by health care personnel without strong medical training. In this context of increasing health care costs, guidelines are at the end of the beginning but, for the expert, they are not the beginning of the end.

## Competing interests

One of us (RDL) has received funds or support from Sanofi-Aventis, Eli Lilly, MSD, Boehringer-Ingelheim, Pfizer and is currently on an Advisory Board of Novo-Nordisk. SAP has no conflicts of interest.

## Authors’ contributions

The authors had an equivalent contribution to this article. Both authors approved the final version of the manuscript.

## Authors’ information

Professor R. David Leslie is Consultant Physician at St Bartholomew’s Hospital and Professor of Diabetes and Autoimmunity at the Blizard Institute, Barts and the London School of Medicine, Queen Mary University of London.

Stavroula A. Paschou, MD, PhD has worked as an EASD Albert Renold Fellow at the Department of Diabetes, St Bartholomew’s Hospital and Blizard Institute, Barts and the London School of Medicine, Queen Mary University of London.

## Pre-publication history

The pre-publication history for this paper can be accessed here:

http://www.biomedcentral.com/1741-7015/11/161/prepub
